# Ambient Mercury Observations near a Coal-Fired Power Plant in a Western U.S. Urban Area

**DOI:** 10.3390/atmos10040176

**Published:** 2019

**Authors:** Lynne E. Gratz, Chris S. Eckley, Story J. Schwantes, Erick Mattson

**Affiliations:** 1 Colorado College, Environmental Studies Program, Colorado Springs, CO 80903, USA;; 2 U.S. Environmental Protection Agency, Region 10, Seattle, WA 98101, USA;; 3 Colorado Department of Public Health and Environment, Air Pollution Control Division, Glendale, CO 80246, USA;

**Keywords:** urban, diurnal, power plant, legacy mercury, re-emission, surface flux

## Abstract

We report on the continuous ambient measurements of total gaseous mercury (TGM) and several ancillary air quality parameters that were collected in Colorado Springs, CO. This urban area, which is located adjacent to the Front Range of the Rocky Mountains, is the second largest metropolitan area in Colorado and has a centrally located coal-fired power plant that installed mercury (Hg) emission controls the year prior to our study. There are few other Hg point sources within the city. Our results, which were obtained from a measurement site < 1 km from the power plant, show a distinct diel pattern in TGM, with peak concentrations occurring during the night (1.7 ± 0.3 ng m^−3^) and minimum concentrations mid-day (1.5 ± 0.2 ng m^−3^). The TGM concentrations were not correlated with wind originating from the direction of the plant or with sulfur dioxide (SO_2_) mixing ratios, and they were not elevated when the atmospheric mixing height was above the effective stack height. These findings suggest that the current Hg emissions from the CFPP did not significantly influence local TGM, and they are consistent with the facility’s relatively low reported annual emissions of 0.20 kg Hg per year. Instead, variability in the regional signal, diurnal meteorological conditions, and/or near-surface emission sources appears to more greatly influence TGM at this urban site.

## 1. Introduction

Mercury is a neurotoxin that can severely impact cognitive abilities, particularly in a developing nervous system or at high levels of exposure [1]. Elevated levels of methylmercury, which is the organic and more toxic/bioaccumulative form of Hg, have been reported in numerous bird, fish, and mammal species [2], and the primary pathway for human exposure is through the consumption of contaminated fish [1]. Mercury is largely introduced to terrestrial and aquatic ecosystems through atmospheric deposition, where it can undergo methylation and bioaccumulate within food chains [2,3].

Atmospheric Hg primarily exists in the elemental vapor phase (gaseous elemental Hg; GEM), which is not highly water soluble and it has an estimated atmospheric lifetime of 0.5 to one year [2]. Mercury is also present in much lower concentrations in an oxidized form, either in the gas phase (gaseous oxidized Hg; GOM) or bound to particles (particle-bound Hg; PBM), which is readily removed by wet and dry deposition [2,3]. Thus, the spatial impacts of Hg emissions on ecosystems and human health range from local to global in scale.

Both natural and anthropogenic processes can emit Hg to the atmosphere. Stationary fuel combustion, gold mining, nonferrous metal smelting, and cement production are the leading anthropogenic sources worldwide [4], while volcanic and geothermal activity are the major natural releases [5]. Following its release to the environment, Hg also undergoes complex biogeochemical cycling. For example, emitted Hg that is not incorporated into food chains May remain in the terrestrial and aquatic ecosystem s as legacy Hg, which is available for eventual re-emission through biomass burning or evasion from the surfaces onto which it was deposited [6]. In the vicinity of Hg point sources, studies have shown the potential for enhancements in both ambient air concentrations [7] and wet deposition [8], while the surface fluxes of legacy Hg can also contribute upwards of 40–50% of ambient gaseous Hg [9]. Previously released Hg can continue to cycle in the environment and prolong the impacts of legacy emissions [10].

**Figure 1.**
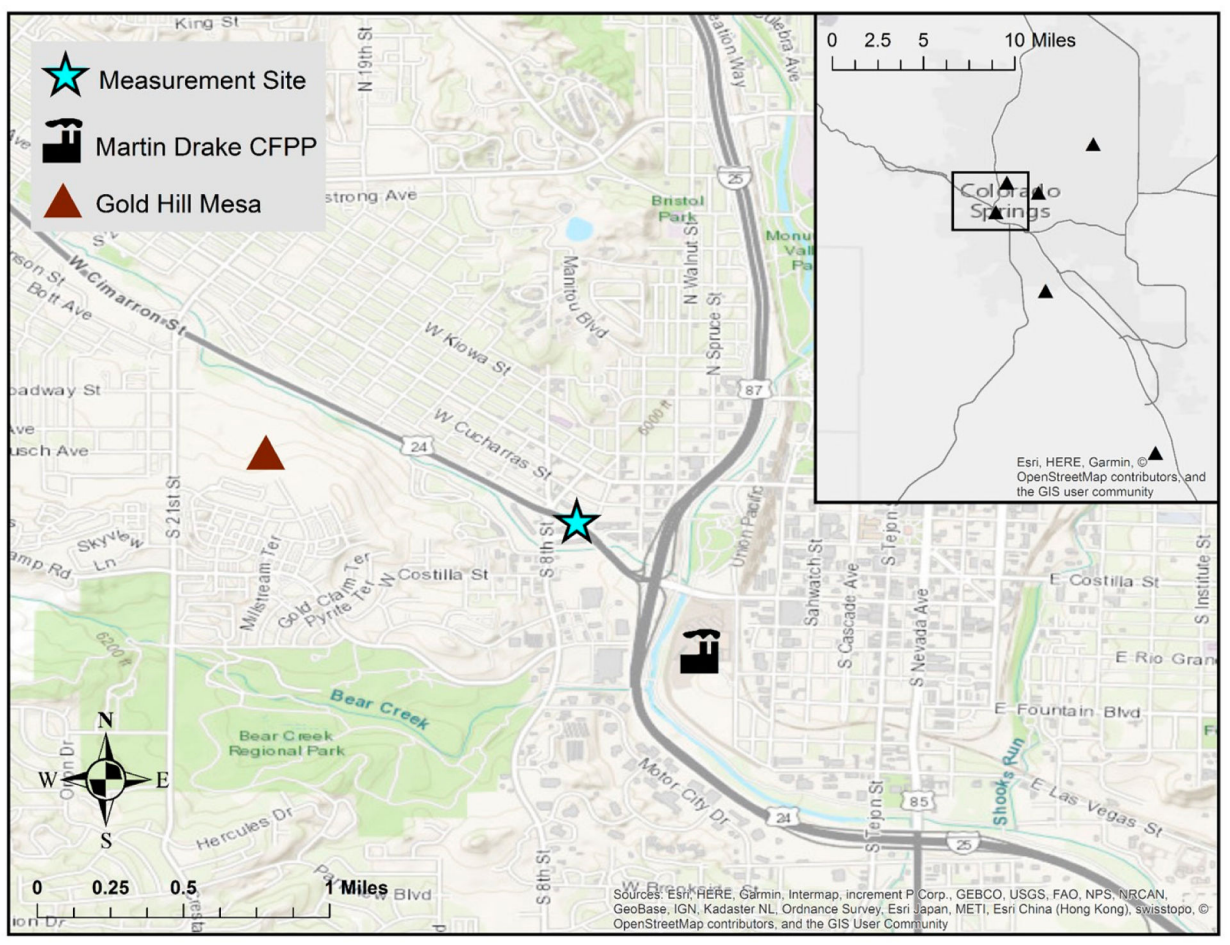
Location of the air quality monitoring site with respect to Interstate-25, Highway 24, and the Martin Drake coal-fired power plant (CFPP). Inset shows Hg point sources emitting > 0.05 kg/year; note that, other than the Ray D. Nixon CFPP (southeast corner; annual emissions 20 kg/yr), all facilities shown have annual Hg emissions between 0.05–0.25 kg/yr [15].

Given that coal combustion has historically been one of the largest sources of atmospheric Hg in the United States (U.S.) [11,12], the characterization of the emission, transport, and fate of power plant Hg emissions has been paramount in the effort to design effective regulations and effectively mitigate ecosystem Hg contamination. In 2011, the United States Environment al Protection Agency (U.S. EPA) issued the Mercury and Air Toxics Standards to reduce pollutant emissions from coal- and oil-fired power plants [13]. These regulations had been effectively pending since the 1990 Clean Air Act Amendments, when contaminants, like Mercury (Hg), were listed as Hazardous Air Pollutants that required further study and mitigation of their impacts on ecosystems and human health. Direct stack emissions of Hg in the U.S. declined by 55% from 2005 to 2015 with the shift to natural gas and the installation of emission controls [12]. Urban airsheds can represent a particularly complex mixture of stationary and point source emissions that are augmented by meteorological patterns that affect the temporal variability in Hg and other pollutant concentrations. The juxtaposition of the major population centers within such airsheds suggests the potential for more direct pollutant exposure pathways from local sources. To that end, this study quantifies the ambient levels of Hg and several ancillary air quality parameters in Colorado Springs, CO, which is the second largest population center in the state (2017 city estimate: 464,474) [14], with a land area of approximately 500 km^2^. In addition to its urban nature, it provides a unique study location due its position along the Front Range of the Rocky Mountains, where diurnal mountain wind patterns affect surface meteorology and boundary layer mixing. The city also has a centrally located coal-fired power plant (CFPP) but few other Hg point sources within the city (Figure 1) [15]. The 185 megawatt Martin Drake CFPP has been in operation for nearly a century, though its two current generating units came online in the late 1960s and early 1970s [16]. The plant provides about one-quarter of the electricity for Colorado Springs [16], but there are concerns in the community over the age of the plant, its impact on local air quality, and emissions of greenhouse gases that contribute to climate change. In November 2015 the Colorado Springs Utility Board voted to decommission Martin Drake no later than 2035, although due to ongoing public pressure, an earlier shutdown is being considered.

There is substantial variability in Hg emissions from fossil fuel electric power generating units; the average (± 1σ) plant releases 23 ± 1.8 kg/yr, but it can range to over 450 kg/yr for some of the largest facilities [17]. The annual emissions from the Martin Drake CFPP are reportedly 0.20 kg Hg/yr, which is on the lower end of the range in typical CFPP Hg emission sources and two orders of magnitude lower than the Ray D. Nixon CFPP (20 kg Hg/yr), a 268 megawatt facility that is located approximately 24 km southeast of Martin Drake (Figure 1) [15]. Earlier U.S. EPA National Emissions Inventories (NEI) document Martin Drake as releasing 0.30 kg Hg/yr (2008 NEI) [18] and 0.26 kg Hg/yr (2011 NEI) [17], though the NEI has been shown to underestimate the CFPP Hg emissions by nearly 40% [19]. In August 2015, prior to our study, halogenated PAC injection Hg emission controls were added to the Drake and Nixon facilities [20]. While annual Hg emissions from the Drake plant are reportedly quite low, as of the 2014 NEI, this facility was the third highest emitter of SO_2_ in the state of Colorado (3.1 × 10^6^ kg/yr), with annual emissions that are comparable to the Nixon facility (3.0 × 10^6^ kg/yr). Sulfur dioxide (SO_2_) emission controls went online at each of Martin Drake’s two operating units in February 2016 (unit 7) and September 2016 (unit 6), whereas the Nixon facility reports SO_2_ scrubbers going online in May 2017 [20].

Herein, we report continuous measurements of total gaseous Hg (total gaseous mercury (TGM) = GEM + GOM), carbon dioxide (CO_2_), SO_2_, carbon monoxide (CO), and meteorological parameters that were collected from June 15 to October 20, 2016 at a site approximately 0.9 km northwest of the Martin Drake power plant (Figure 1). Our objectives are to (1) characterize the temporal variability in ambient Hg levels in this urban airshed, (2) assess whether the centrally-located CFPP with recent emission controls is a significant sources of Hg to the local atmosphere, and (3) consider the contribution of sources other than the CFPP to the ambient Hg level.

## 2. Results

### 2.1. Summary of TGM and Ancillary Parameters

**Figure 2.**
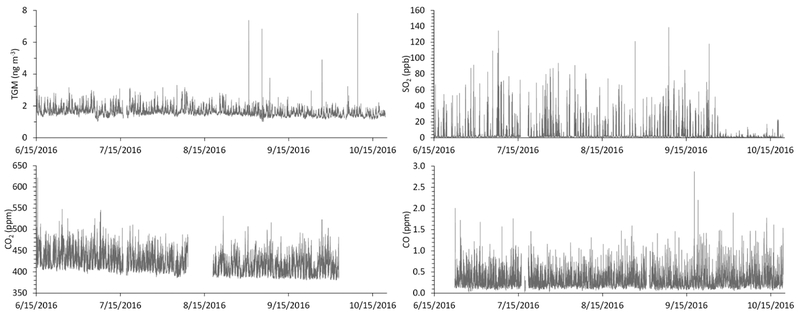
Continuous measurements of (**a**) total gaseous mercury (TGM), (**b**) SO_2_, (**c**) CO_2_, and (**d**) CO at the measurement site; from June 15 to October 20, 2016. TGM data are five-minute integrated measurements. SO_2_, CO_2_, and CO data are five-minute averages of 1-minute measurements.

The average (±1σ) ambient TGM concentration that was measured in our study was 1.6 ± 0.3 ng m^−3^ (Table SI-1). This is on the higher end of the reported range of Northern Hemisphere background concentrations (1.3–1.6 ng m^−3^) [10], but it is on the lower end for North American urban areas (1.6 ± 0.3 – 1.9 ± 0.8 ng m^−3^) [21]. However, the 75th (1.7 ng m^−3^) and 90th (2.0 ng m^−3^) percentiles suggest there are local or regional Hg sources that contribute to elevated TGM at our site. There are four periods of highly elevated TGM that can be statistically characterized as major outliers (Figure 2), and the associated data points (N = 5; TGM > 4.8 ng m^−3^) have been removed in the subsequent analyses; these periods are shown in the Supplemental Information. The aforementioned statistics describing the data distribution are unchanged with the removal of these points.

Sulfur dioxide mixing ratios (3.4 ± 7.5 ppb; Table SI-1) are consistent with a site that is in close proximity to a CFPP, given that fossil fuel combustion directly emits SO_2_ to the atmosphere [22]. The lifetime of SO_2_ against oxidation by the hydroxyl radical to form sulfuric acid (H_2_SO_4_) is approximately one week [22]; thus, we anticipate minimal SO_2_ loss over the very short distance between the power plant stack and the measurement site. There is an observable reduction in the ambient SO_2_ concentrations at our monitoring site; in late September, which is consistent with the aforementioned scrubber installation (Figure 2).

The average CO_2_ mixing ratio (419 ± 22 ppm) is above the 2017 global average (405 ppm) [23] and the data distribution is consistent with a site in close proximity to a CFPP and two major roadways (Figure 1; Table SI-1). The observed CO mixing ratios (0.30 ± 0.18 ppm), which are approximately double the Northern Hemisphere background values that were reported in other studied [24,25], similarly reflecting the near-road nature of the measurement site. One major CO outlier (2.8 ppm on 9/17/1 6; Figure 2) has been removed in the sub sequent analyses.

Using hourly-averages of the five-minute observations, we find that TGM is significantly positively correlated with CO_2_ (R = 0.62, p < 0.001) and relative humidity (R = 0.43, p < 0.001), but it is negatively correlated with wind speed (R = −0.40, p < 0.001) (Table SI-2). Interestingly, TGM and SO_2_ are uncorrelated (R = −0.06, p = 0.002); instead, SO_2_ is positively correlated with temperature (R = 0.28, p < 0.001) and wind speed (R = 0.36, p < 0.001), but not strongly correlated with any of the other chemical measurements. We observe a weak positive correlation between CO and CO_2_ (R = 0.44, p < 0.001), which is consistent with a shared motor vehicle emissions source at this urban measurement sits. Other potential sources or CO_2_, including the power plants and surface fluxes (e.g., soil respiration), likely weaken the correlation.

### 2.2. Directionality

**Figure 3.**
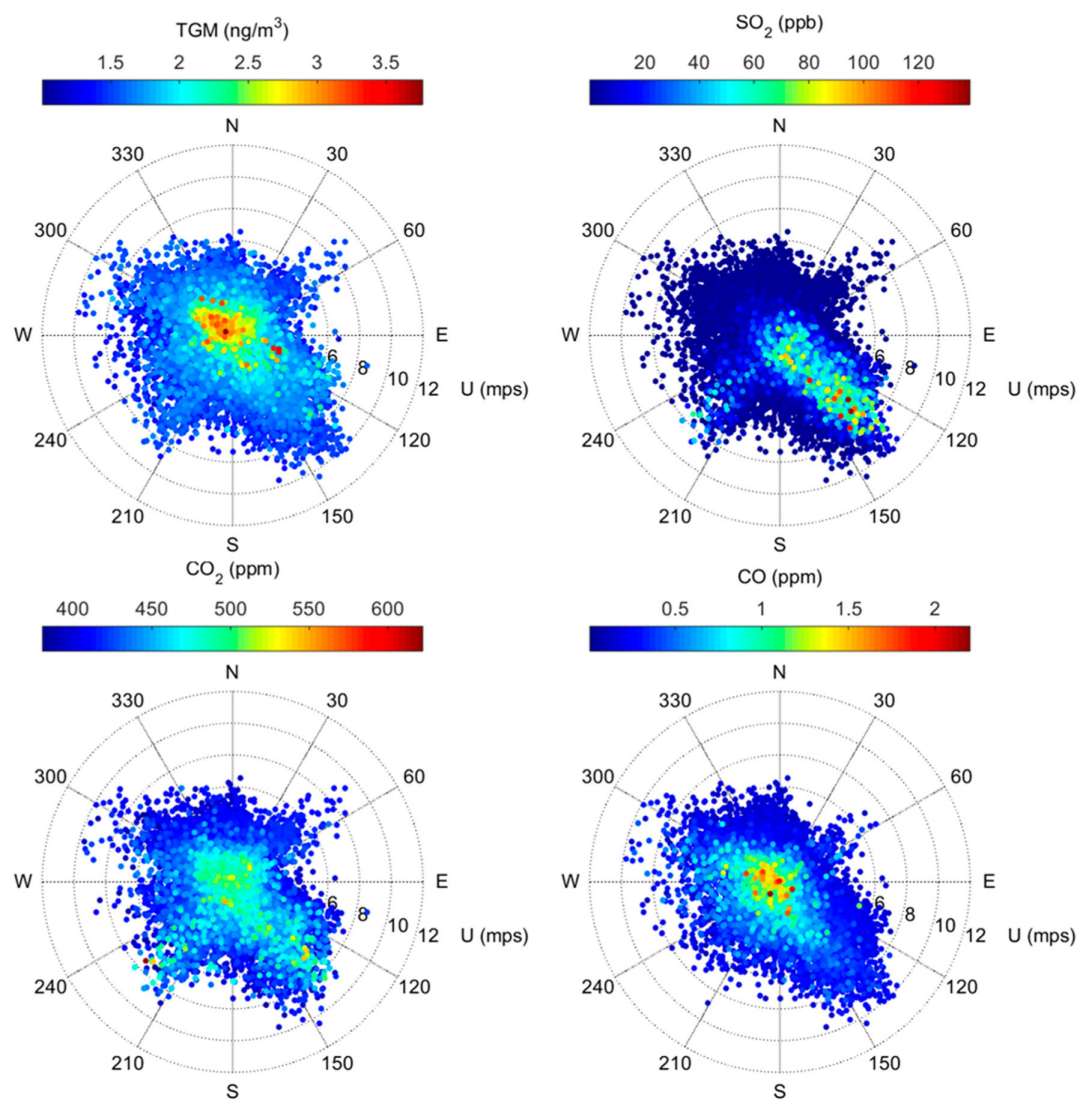
Polar plots of 5-minute TGM, SO_2_, CO_2_, and CO observations at the monitoring site, June 15–October 20, 2016.

Figure 3 displays the relationship between five-minute measured concentrations and wind speed/direction. We find that elevated SO_2_ mixing ratios are very strongly associated with winds from the southeast centered around 135°, which is in line with the position of the nearby Martin Drake CFPP, as well as the more distant Ray D. Nixon plant relative to the measurement site (Figure 1). Under southeasterly winds (112.5°–157.5°), the mean of five-minute SO_2_ mixing ratios was 8.5 ± 3.0 ppb, which was more than double the mean mixing ratios measured within the other cardinal and inter-cardinal wind directions. Moderately elevated SO_2_ mixing ratios were also associated with south-southwesterly winds (south: 3.9 ± 1.6 ppb; southwest: 3.4 ± 1.4 ppb), which May be attributed to the Victor gold mine in Cripple Creek, CO (approximately 30 km southwest of our measurement site; SO_2_ emissions 2.8 × 10^4^ kg/yr [15]). These directional patterns, along with the observed decline in ambient SO_2_ in late September 2016 with the installation of an SO_2_ scrubber at the Martin Drake CFPP, confirm that emissions from the Drake plant are detected at the measurement site;, even when considering their very close proximity to one another, which could result in emissions passing over the site, rather then being mixed down to the surface.

In contrast, elevated TGM concentrations typically occurred under low wind speeds (Figure 3, Table SI-2). This behavior is somewhat similar to CO, for which the association with low wind speed is consistent with motor vehicle emissions along the adjacent Interstate-25 arid Highway 24 roadways. However, TGM and CO are not significantly positively correlated (Table SI-2), which suggests that motor vehicle emissions are not a significant source of TGM at this site. While low levels of Hg are present in vehicle fuels and are emitted during combustion [26,27], in general, mobile sources are typically very small relative to stationary sources like CFPPs [15]. TGM was significantly higher under northwesterly (292.5°–337.5°) and northerly (337.5°–22.5°) flow, where the mean concentrations were 1.7 ± 0.3 ng m^−3^. We also find the strongest correlation between TGM and CO_2_, under winds that range from northwesterly to northeasterly (R = 0.59 to 0.65, p < 0.001).

### 2.3. Temporal Variability

**Figure 4.**
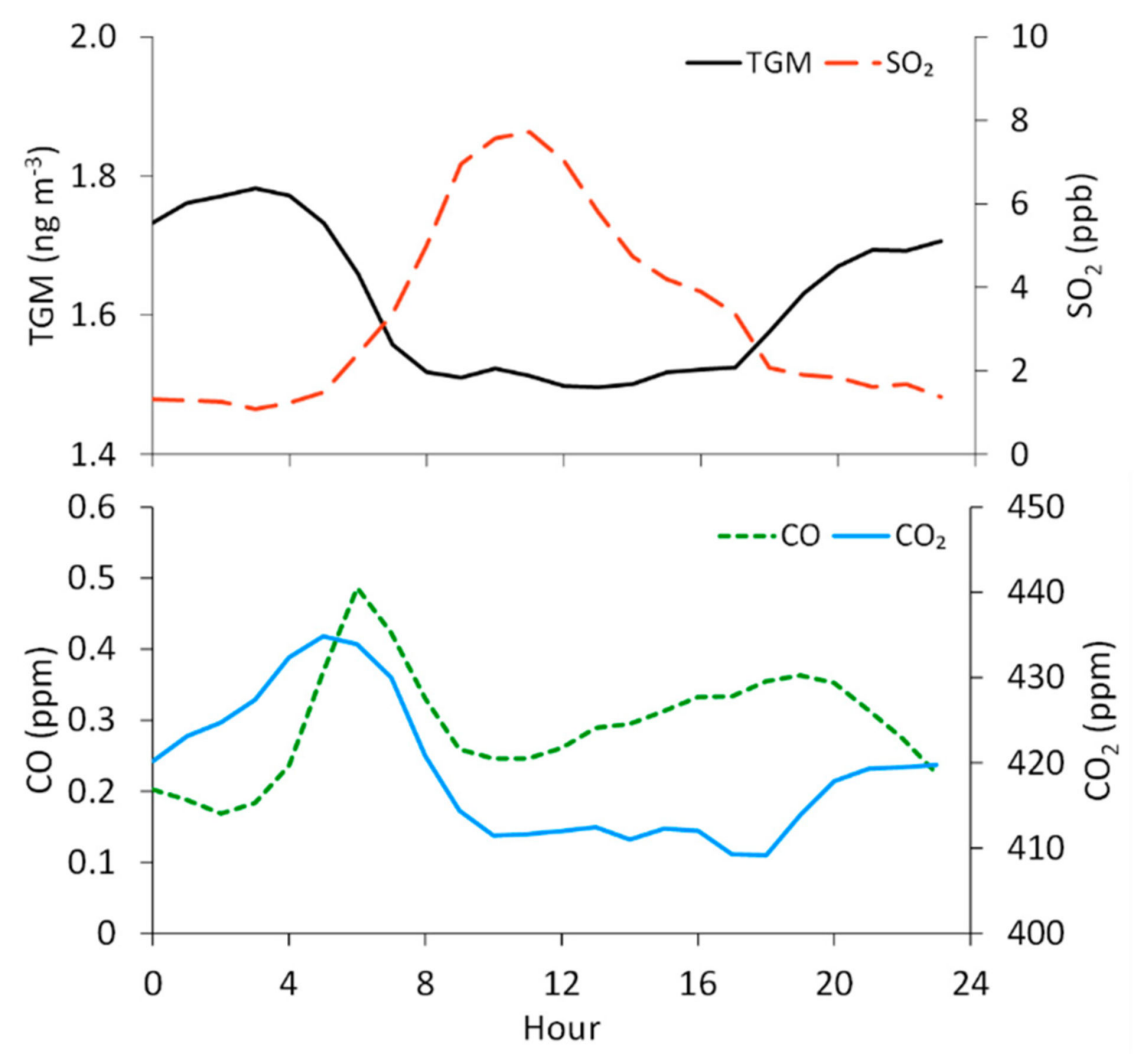
Overall hourly-averaged concentrations of TGM and mixing ratios of SO_2_, CO, and CO_2_ at the measurement site. Hours are in Mountain Standard Time (MST), and the averages are assigned to the starting hour of each 1 h period. The means of each hourly standard deviation (not shown graphically) are 0.2 ng m^−3^, 6.1 ppb, 0.16 ppm, and 20 ppm for TGM, SO_2_, CO, and CO_2_, respectively.

We find that TGM concentrations are the highest at night and in the early morning, with the maximum on average occurring at 3:00 MST (1.8 ± 0.3 ng m^−3^) and the minimum at 13:00 MST (1.5 ± 0.1 ng m^−3^) (Figure 4). Defining nighttime as 19:00–7:00 and daytime as 7:00–19:00, we find significantly different mean TGM concentrations of 1.7 ± 0.3 ng m^−3^ and 1.5 ± 0.2 ng m^−3^, respectively. In contrast, SO_2_ displays a midday maximum that appears to be strongly influenced by diel variability in wind direction, given that the southeasterly flow dominated during daytime hours (8:00–16:00 MST, 25–40% of hourly-averaged observations), while northwesterly winds were more frequent in the late night and early morning hours (20:00–5:00 MST; ~40% of hourly-averaged observations). We find the diel TGM pattern to be similar to that of CO_2_, which displays a morning maximum at 5:00 (435 ± 26 ppm) and low levels at midday, with a minimum at 18:00 (409 ± 14 ppm). The diel variability in CO_2_ can be attributed, in part, to rush hour motor vehicle emissions, as is evident in the diel pattern for CO (Figure 4), but also to soil respiration, which would be the dominant surface flux in the evening and early morning in the absence of photosynthesis.

**Figure 5.**
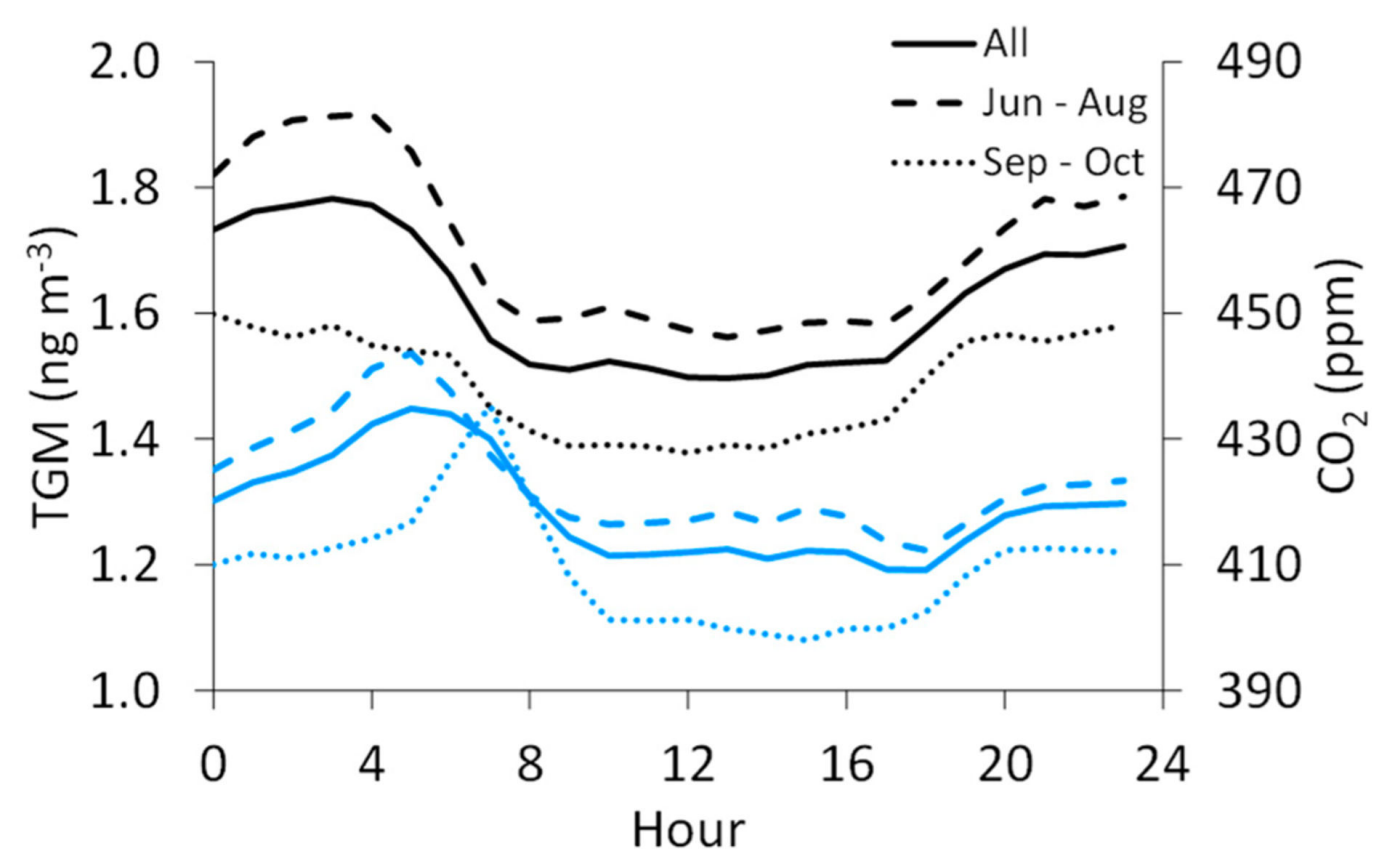
Diel patterns for TGM (blade) and CO_2_ (blue) for the full dataset (solid line), June through August (long; dashed line), and September through October (short dashed line).

We also consider the summer-fall variability in TGM and the other measured species, though the summer months (June–August) contain approximately 1.5 times more data for TGM, SO_2_, and CO than in fall (September–October), while for CO_2_, there is about two times more summer data. Nevertheless, summertime average TGM concentrations (1.7 ± 0.3 ng m^−3^) are significantly higher than in fall (1.5 ± 0.2 ngm^−3^). The average CO_2_ mixing ratio is also significantly higher in summer (423 ± 21 ppm) than in fall (410 ± 19 ppm). The diel pattern for TGM in fall shifts to lower mean values with reduced variability around the mean (day: 1.4 ± 0.1 ng m^−3^; night: 1.6 ± 0.2 ng m^−3^) when compared to summer (day: 1.6 ± 0.2 ng m^−3^; night: 1.8 ± 0.3 ng m^−3^), though the timing for the daytime minimum and nighttime maximum concentrations remains similar (Figure 5). For CO_2_, the daily maximum shifts to) a later hour in fall (7:00 MST) than in summer (5:00 MST). The summer-fall differences are less pronounced for SO_2_ (summer: 3.9 ± 8.2 ppb, fall: 2.7 ± 6.3 ppb) and CO (summer: 0.29 ± 0.16 ppm, fall. 0.31 ± 0.21 ppm), though the means for both species are significantly different by season.

## 3. Discussion

**Figure 6.**
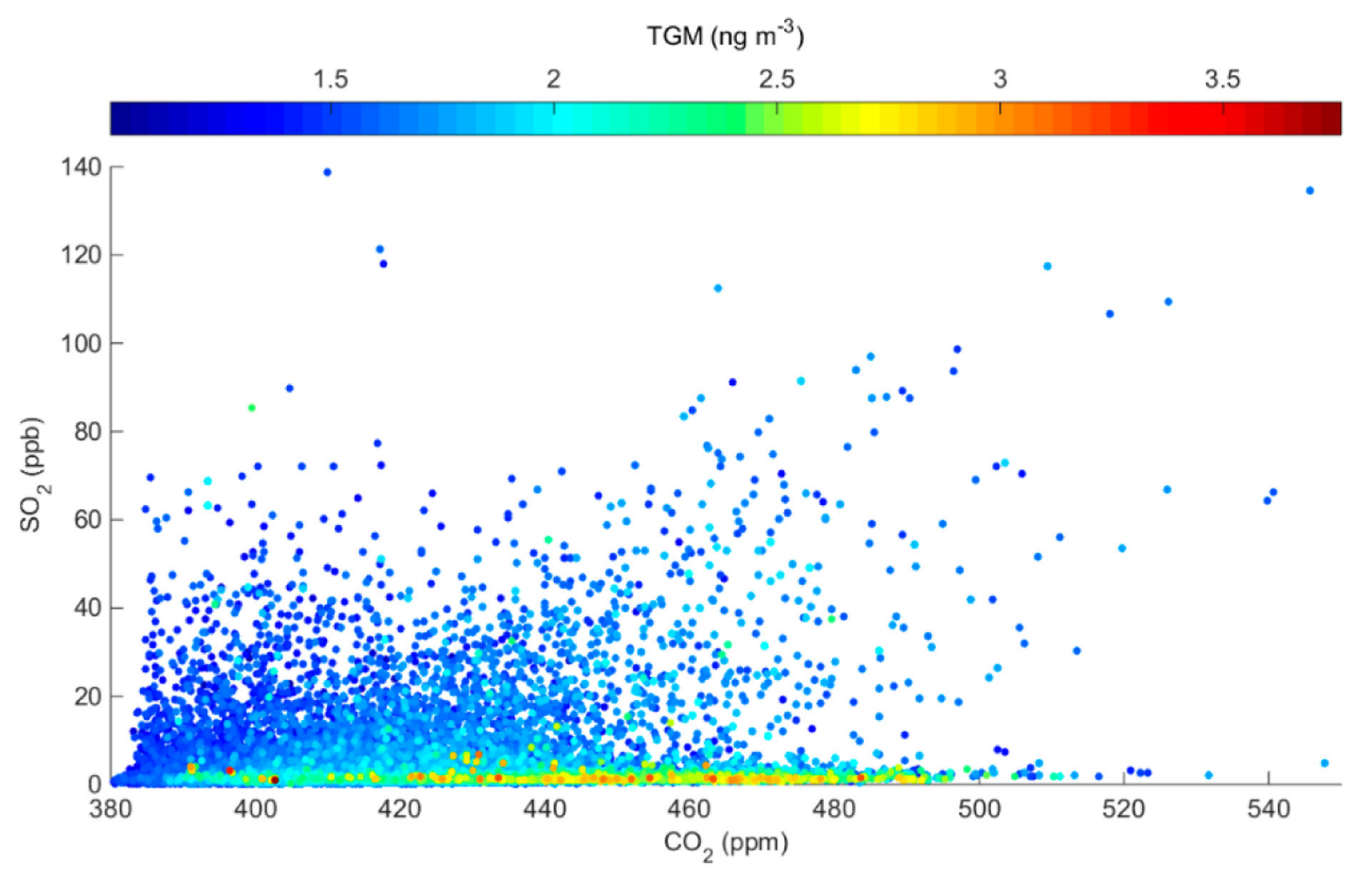
Observed SO_2_ versus CO_2_ at the monitoring site, color-coded by corresponding TGM measurements.

To specifically identify whether the observed variations in TGM are related to releases of Hg from the adjacent Martin Drake CFPP, we first consider results of a Hybrid Single-Particle Lagrangian Transport (HYSPLIT) dispersion model [28] that was initiated from the plant during; periods when the measurement site was likely impacted by CFPP emissions (determined by persistent southeasterly flow and enhanced SO_2_; see Section 4.4). During all of the simulations, the modeled concentrations are < 5 pg m^−3^ and are thus below the method detection limit of the Tekran 2537A. This finding can be attributed to the relatively low reported annual Hg emissions for Martin Drake in the 2014 NEI. Even if those; reported emissions are increased in tire model by 39%, following the assessment by Ambrose et al. [19] regarding the NEI’s underestimation of CFPP Hg emissions, the resulting concentrations at our measurement site would still be too low to be defected. To further demonstrate the lack of influence of current emissions from the Martin Drake CFPP on our measured TGM concentrations, we examine the relationships between SO_2_, CO_2_, and TGM given that all three species could be plausibly attributed to CFPP emissions (Figure 6). In general, we find that the largest TGM enhancements were often detected during periods when SO_2_ was extremely low; more specifically, for the 90^th^ percentile TGM concentrations, the mean (± 1σ) SO_2_ was only 1.8 ± 3.8 ppb in comparison with the study mean (3.4 ± 7.5 ppb) and mean levels that were detected under southeasterly flow from the direction of the plant (8.5 ± 3.0 ppb; Figure 3). These findings, together with the previously discussed correlation and directional analyses, suggest that sources other than the local CFPP are likely contributing to the observed distribution of TGM concentrations.

The diel TGM pattern in our study differs from observations from rural/background sites where the minimum concentrations typically occur pre-dawn and maximum concentrations occur in the afternoon [21,29]. These diel dynamics have been attributed to nighttime TGM deposition in the stable nocturnal boundary layer, followed by daytime surface emissions. The opposite pattern, one that is more similar to what we report here, has been observed at several urban/industrial monitoring sites and it is proposed to be caused by high surface concentrations that are diluted with daytime warming and enhanced vertical mixing [21,30–34]. Here, we consider the effect of diurnal changes in atmospheric stability and mixing height on our TGM observations, which can aid in identifying the relative importance of point source or near-surface emissions [9]. In general, mixing heights often show diurnal variations, with neutral/stable atmospheric conditions occurring at night (low or no discernible mixing height) and unstable conditions (higher mixing height) developing as the surface temperatures increase during the day. Following similar methods to those described by Eckley et al. [9] and using the stack height of Martin Drake as a reference, we find that TGM concentrations were slightly higher when the mixing height was closer to the surface (below the stack height) when compared to when it was higher and above the stack height (mixing height below stack height: 1.62 ± 0.16 ng m^−3^, n = 31; mixing height above stack height 1.56 ± 0.13 ng m^−3^, n = 95; t-test p-value = 0.03). We obtain similar results when using the effective stack height rather than the actual stack height in the analysis (below: 1.60 ± 0.14 ng m^−3^, n = 51; above: 1.56 ± 0.13 ng m^−3^, n = 75); however, the differences were not significant (p = 0.9). Similar to TGM, CO_2_ was also higher when the mixing height was lower than the effective stack height (below: 413 ± 14 ppm, n = 46; above: 408 ± 10 ppm, n = 55; t-test p-value=0.04).

These results are not only consistent with the lack of contribution from current Martin Drake CFPP emissions on observed TGM, but they also suggest that the variations in the TGM concentrations may be driven by surface/near-surface emissions and/or variability in other regional source emissions (i.e., larger point sources along the Colorado Front Range) that are mixed down to the surface under nighttime neutral/stable conditions. In general, solar radiation is considered to be a major determinant in driving Hg from various surface materials (e.g., soil, vegetation, or pavement), suggesting that the greatest flux of Hg back to the atmosphere would be observed during daytime hours and during the summer season [35]. resulting in lower ambient concentrations; in contrast, the concentrations are higher at night when the mixing height is lower and the mixed layer is more stable, containing surface emissions that are close to their source [36]. We similarly calculate that the mixing height is significantly higher during the day than it is at night for our measurement site, and we therefore propose that this might result in greater daytime mixing and the dilution of surface emissions within the atmosphere and a concentration of TGM toward the surface at night. Comparing the summer and fall mixing heights also show s that the mixing height was more often above the stack height during the fall (40% of the time) than during the summer (24% of the time; *t*-test p = 0.004), which, when considering that TGM concentrations were significantly higher in the summer than the fall further, suggests that variables affecting surface air emissions, such as solar radiation and temperature, may have a larger influence on the TGM concentrations than local point source emissions.

In general, surface Hg fluxes could originate from the re-emission of previously deposited Hg, geologically enriched soils, or anthropogenically disturbed landscapes. We point out that the Interstate-25–Highway 24 interchange adjacent to our site was under construction throughout our study, and thus large swaths of exposed soil May have contributed to local surface Hg fluxes.

In addition to previously deposited Hg from local or regional sources in the Colorado Front Range, one potentially interesting source of surface Hg flux that is adjacent to our measurement site is a 400-acre abandoned gold and silver mine tailings area (Gold Hill Mesa), approximately 1 km west-northwest of the monitoring site (Figure 1) [37], which May contain elevated levels of Hg and could contribute to elevated surface emissions [38]. Other potential sources of Hg in urban areas could include relatively low-level non-point emissions from buildings, waste centers, hospitals/dental facilities, or roads [39–41].

We lastly consider tropospheric mixing as a potential source of elevated TGM. Due to our monitoring location being in a relatively high elevation environment that is susceptible to the upslope/downslope mountain wind patterns, there is the potential for this location to be impacted by the pool of oxidized Hg-enriched free tropospheric air that has been predicted for the U.S. Southwest in chemical models and observed in recent studies [42–44]. Deep upper air intrusions can transport air from the free troposphere or stratosphere, which impacts surface air com position, particularly in the Western U.S.; however, these events are largely confined to springtime when regional meteorology supports this type of vertical transport [45]. Low relative humidity and reduced levels of boundary layer pollutants likely characterize an influx of free tropospheric air, whereas in our dataset, we find positive relationships between TGM, relative humidity, CO_2_, and CO (Table SI-2). Furthermore, when we consider hourly ozone measurements from the closest CDPHE air monitoring station (Manitou Springs, ~ 6 km northwest of monitoring site, elevation 1960 m), we also find that ozone and TGM are not strongly correlated (R = −0.06, p < 0.05). Thus, we propose that free tropospheric subsidence of air enriched in oxidized Hg is not a major source of the elevated levels of TGM that we observe in this summer/fall study.

Overall, our study used multiple lines of evidence to determine whether a proximate CFPP was having a discernable impact on the local TGM concentrations. The results of the directional analysis, correlations with other air quality parameters (SO_2_, CO_2_), plume model, and mixing height analysis provide a consistent conclusion that an impact from current CFPP emissions could not be identified and it is consistent with the low Hg emissions that were reported in the NEI. Instead, periods of elevated TGM appear to be more related to surface and/or near-surface emissions, but in the absence of additional measurements, it is difficult to conclusively distinguish between the potential surface sources. Given that soils are considered to be the largest Hg pool in the terrestrial environment in part due to increased loadings from anthropogenic activities, yet atmosphere-terrestrial interactions are one of the least constrained processes in the global Hg cycle [10], future work to characterize surface emissions and re-emissions will further advance our understanding of Hg biogeochemical cycling and the fate of Hg in the ecosystem.

## 4. Materials and Methods

### 4.1. Site Description

We measured TGM and the ancillary parameters (SO_2_, CO, CO_2_, meteorology) at the Colorado Department of Public Health and Environment (CDPHE) Highway 24 ambient air quality monitoring station from June 15 to October 20, 2016. The site is approximately 0.9 km northwest of the Martin Drake CFPP, 10 m north of Colorado State Highway 24, and 400 m west of Interstate-25 (Figure 1). We selected this site given its urban nature and its proximity to the Martin Drake CFPP, which is the point source of interest for our scientific objectives. Prior measurements at the site confirmed an impact from the Drake facility through the strong dependence of SO_2_ mixing ratios on southeasterly winds from the direction of the plant (as similarly demonstrated in Figure 3).

### 4.2. Ambient Air Measurements

The TGM (GEM + GOM) inlet consisted of a 0.1 micron 47 mm PTFE particulate filter that was housed in a Teflon filter pack and covered by a rain shield. The inlet was connected to 4 m of ${\frac{1}{4}}^{\prime \prime }$ Teflon sample line that was wrapped in Nomex ®insulation and maintained at a temperature of 100 °C. The heated Teflon sample line was connected to a pyrolyzer inside the measurement shelter that consisted of a glass tube (a regenerable particulate filter (RPF) commonly used with the Tekran 2537/1130/1135 speciation system) packed with quartz chips and quartz wool, which was contained in a Lindberg Blue tube furnace heated to 500 °C. We quantified TGM concentrations in integrated five-minute periods using a Tekran 2537A Mercury vapor analyzer. We verified the Tekran 2537A internal permeation source in the laboratory prior to field deployment using manual injections of Mercury vapor from a Tekran 2505 external calibration unit, and it conducted weekly internal permeation source calibrations throughout the four-month study. We independently analyzed the raw Tekran data using the peak height analysis that was described by Swartzendruber et al. [46].

In the absence of reliable commercially-available instrumentation to segregate continuously-measured ambient Mercury species [47], we elected to filter out particulate-bound Mercury and the sample only the gas-phase. Given the site’s proximity to major roadways that were under construction during our measurement period, we also w anted to reduce the risk that coarse particulate matter might contaminate the sample line. We acknowledge that the inlet filter May also remove some GOM, due to its reactive nature, and so most of the TGM that was measured is likely to be GEM [48]. Given that both the Martin Drake and Ray D. Nixon CFPPs operate bag houses to remove particulate emissions, we also anticipate any Hg emissions from the nearby power plants to be predominantly gaseous. We acknowledge that gaseous oxidized Hg can readily partition to particles via condensation, adsorption, or chemical reactions if particulate matter is present in the ambient atmosphere [49], and thus it is possible that our prioritization of gaseous Hg May underestimate total Hg levels in the ambient atmosphere. Though CDPHE does not measure PM_2.5_ at the Highway 24 site, hourly PM_2.5_ measurements are available from a nearby air monitoring site (Colorado College) approximately 2 km northwest of the Highway 24 site. During our measurement period (June 15-October 20, 2016), the mean (± 1σ) hourly PM_2.5_ concentration at Colorado College was 6.8 ± 3.1 μg m^−3^, which is below the primary one-year NAAQS (12.0 μg m^−3^). The 98th percentile for the same period was 14 μg m^−3^, which was also below the primary 24-hour NAAQS (35 μg m^−3^). For reference, cities in the U.S. with populations between 500,000 and 1,000,000 report mean (± 1σ) hourly PM_2.5_ concentrations of 8.8 ± 2.5 μg m^−3^ at the sites closest to those urban areas [50], placing Colorado Springs on the lower end of urban PM_2.5_ levels.

We measured CO_2_ using a LiCor LI-840A continuous CO_2_/H_2_O analyzer with external pump that pulled air through ${\frac{1}{4}}^{\prime \prime }$ O.D., 1/8″ I.D. Bev-A-Line polyethylene tubing. The inlet was fitted with a Millipore-FA filter unit containing a 1.0 μm PTFE filter. We logged measurements with one-minute frequency and averaged them to five-minutes to match the Tekran data. We note that the CO_2_ measurements concluded on October 3 due to instrument relocation for another project.

The CDPHE Air Pollution Control Division continuously measures CO, SO_2_, temperature, wind speed, wind direction, and relative humidity at the Highway 24 site, which we incorporated for our time period of interest. Carbon monoxide is measured with a Thermo Scientific 48i analyzer and SO_2_ is measured with a Teldyne API 100E analyzer. Inlet heights are 4–6 m above ground and 1 m above and away from the shelter, in accordance with EPA guidelines. The CDPHE provided one-minute measurement data, from which we computed five-minute averages to match the TGM measurements. Calibration and QA/QC procedures follow the CDPHE Quality Assurance Project Plan (QAPP) [51]. Certified hourly data from this and other CDPHE air monitoring stations, including the Manitou Springs and Colorado College stations that were mentioned in this manuscript, are available through the U.S. EPA Air Quality System (AQS) database (https://aqs.epa.gov/api).

### 4.3. Data Analysis

We analyzed the data from this study while using a combination of IBM SPSS Statistics v22, Matlab vR2014b, and Microsoft Excel 2013. Correlations are given using Pearson correlation coefficients. Statistical significance is reported for p-values less than 0.05, unless otherwise specified. Unless noted, all data analyses utilize the five-minute integrated or averaged values from the dataset.

### 4.4. HYSPLIT Plume Modeling

We used the Hybrid Single-Particle Lagrangian Transport (HYSPLIT) model [28] to simulate plume dispersion from the Martin Drake CFPP. Our objective was to determine whether Hg emissions from the plant would be detectable at the air monitoring site given the low reported annual Hg emissions from the plant (0.20 kg/yr) and the average method detection limit of 0.06 ng m^−3^, as reported in other studies for Tekran 2537A GEM measurements [52,53]. We used the latitude, longitude, stack height, and the NEI-reported Hg emission rate of Martin Drake for plume initialization. We assumed continuous emission and thus converted the annual Hg emission rate to an hourly rate of 0.023 g/hr. We set the top of model to 500 m AGL and chose a sampling level of 100 m AGL. For the model runs, we selected five two-hour periods (one during each month of the study) when the site experienced predominantly southeasterly winds (112.5°−157.5°) and the average SO_2_ mixing ratio was at least 10 ppb. We initialized the emission one hour prior to the two-hour sampling period of interest and produced model output every five minutes to match the Tekran sampling interval. We did not include wet or dry deposition in the model given the very short distance between our source and receptor locations; moreover, the purpose of this model run was not to reproduce our observations, but rather to confirm whether the relatively low annual Hg emissions from the plant would be detectable at our receptor location. After each model simulation, we viewed the output as an overlay in Google Earth to confirm that the modeled plume did, in fact, pass over our receptor site. We used the convert-to-station function with a nearest neighbor interpolation method in HYSPLIT to extract the modeled five-minute concentrations at the air monitoring site during the two-hour period of interest. In all five cases, the two-hour average of modeled concentrations within the plume at our receptor location was less than 5 pg m^−3^ and thus below the Tekran 2537 detection limit. This finding remained true, even if we scaled up the emission rate by 39% following the findings of Ambrose et al. [19] for the NEI’s underestimate of U.S. CFPP Hg emissions.

### 4.5. Mixing Height Analysis

We performed an atmospheric mixing height analysis to identify the relative importance of point source or near-surface emissions on the observed TGM concentrations following similar methods described in Eckley et al. [9]. In summary, we estimated atmospheric stability while using twice-daily sounding data from Stapleton, CO (the nearest National Weather Service upper air sounding location, approximately 100 km north of the measurement site). We consider the sounding at 6:00 local time to represent nighttime conditions (wherein >99% of the data had neutral/stable conditions) and the 18:00 sounding to represent the more unstable conditions in the summertime late afternoon (wherein the mixing height was found to be below the effective stack height 40 % of the time and above the stack height 60% of the time). The virtual potential temperature (VPT) measurements were plotted against the height above ground to determine the mixing height level. The mixing height was compared to the effective stack height to determine the measurement periods when stack emissions May have influenced the monitoring station. The effective stack height was estimated by adding the stack height (76.2 m) [54] and the vertical plume rise, which was calculated while using the stack’s inside diameter, exit velocity, and temperature [54], as well as the ambient temperature and wind speed (following a normalized plume rise equation). On average, the calculated plume rise was 190 m, but it varied between 49 and 749 m above stack height, depending on the conditions, and it is a more accurate comparison to the mixing height.

We considered the mean TGM and other pollutant concentrations for the encompassing 3 h period that is associated with each sounding (i.e., we used the mean TGM concentration between 5:00 and 8:00 for the 6:00 sounding, and between 17 and 20:00 for the 18:00 sounding). The TGM and CO_2_ data were normally distributed (Shapiro–Wilks p < 0.05), and thus parametric statistics were used. To remove the influence of diel variables (e.g., solar radiation, temperature) other than the mixing height influencing TGM concentrations, we only performed the statistical analysis for measurements during the 18:00 sampling period.

## Supplementary Material

SI
