# Supplementary material for: Ambient Mercury Observations near a Coal-Fired Power Plant in a Western U.S. Urban Area
Source: Atmosphere (Basel). Author manuscript; Available in PMC 2020 Jan 1. (PMC6711202; doi:10.3390/atmos10040176)
Supplement: SI [file NIHMS1042652-supplement-SI.pdf]

Supplemental Information for:

Ambient mercury observations near a coal-fired power plant in a western U.S. urban area

Lynne E. Gratz[1], Chris S. Eckley[2], Story J. Schwantes[1], and Erick Mattson[3]

[1]Colorado College, Environmental Studies Program, Colorado Springs, CO, 80903, USA
[2]U.S. Environmental Protection Agency, Region 10, Seattle, WA, 98101, USA
[3]Colorado Department of Public Health and Environment, Air Pollution Control Division, Glendale, CO, 80246, USA

2 Tables
4 Figures

Data Summary

Table SI-1. Summary statistics for 5-minute observations of TGM, SO_2, CO and CO_2 at the measurement site from June 15 to October 20, 2016.

	**TGM (ng m^{-3})**	**SO_2 (ppb)**	**CO (ppm)**	**CO_2 (ppm)**
Mean	1.6	3.4	0.30	419
Median	1.6	1.3	0.24	416
Std. Deviation	0.3	7.5	0.18	22
Minimum	1.0	0	0.01	380
Maximum	7.8	139	2.87	622
N	35963	34355	32026	28858

Table SI-2. Pearson correlation coefficients for hourly-averaged continuous 5-minute measurements. Values in bold are statistically significant at $p < 0.01$, with the exception of the CO-temperature relationship which is significant at $p < 0.05$; all non-bold values are not significant at $p < 0.05$.

	**TGM**	**SO_2**	**CO**	**CO_2**	**Temp**	**RH**	**WS**
**TGM**	1	**-0.06**	**0.16**	**0.62**	**-0.13**	**0.43**	**-0.40**
**SO_2**	--	1	0.00	**0.08**	**0.28**	**-0.11**	**0.36**
**CO**	--	--	1	**0.44**	**-0.05**	-0.03	**-0.22**
**CO_2**	--	--	--	1	**-0.11**	**0.21**	**-0.31**
**Temp**	--	--	--	--	1	**-0.64**	**0.38**
**RH**	--	--	--	--	--	1	**-0.36**
**WS**	--	--	--	--	--	--	1

TGM outliers

On four occasions during the second half of the sampling period we detected TGM concentrations greater than 4 ng m^{-3} that are major statistical outliers in the broader dataset (Figure 2). These four periods are summarized in Table SI-2 with regards to the observed chemical and meteorological patterns. Overall we are unable to identify specific source(s) for each enhancement (in part due to missing ancillary chemical data during two of the four periods), nor can we rule out the possibility of sampling artifacts. The patterns observed do not appear consistent with the broader findings of the larger dataset described in the manuscript. However, we provide graphical depictions below for the four periods (Figures SI-1 – SI-4) for the sake of a complete presentation of the measurements from this study.

Table SI-2. Summary of observations from 20 minutes before and after each of four TGM enhancements > 4 ng m^{-3}. Reported values are the maximum of the 5-min TGM and 1-min SO_2, CO, and CO_2 measurements and the average value for the meteorological measurements.

Date	Time	Max TGM (ng m^{-3})	Max SO_2 (ppb)	Max CO (ppm)	Max CO_2 (ppm)	Avg WS (m/s)	Avg Temp (°C)	Avg RH (%)
8/31/2016	12:15 - 12:55	7.4	N/A	N/A	410	3.0	22.4	52.0
9/5/2016	8:00 - 8:40	6.8	76	0.46	441	2.0	20.4	36.1
9/27/2016	6:05 - 6:45	4.9	5.4	1.65	504	0.54	7.48	61.6
10/10/2016	6:30 - 7:10	7.8	5.8	1.68	N/A	0.62	12.0	43.5

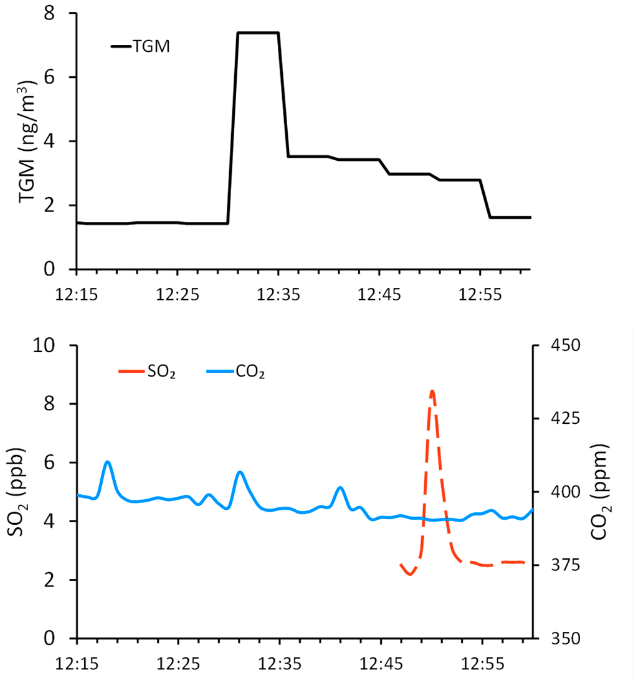

Figure SI-1. 5-min TGM and 1-minute SO_2, CO, and CO_2 measurements on August 31, 2016 from 12:15 MST to 13:00 MST.

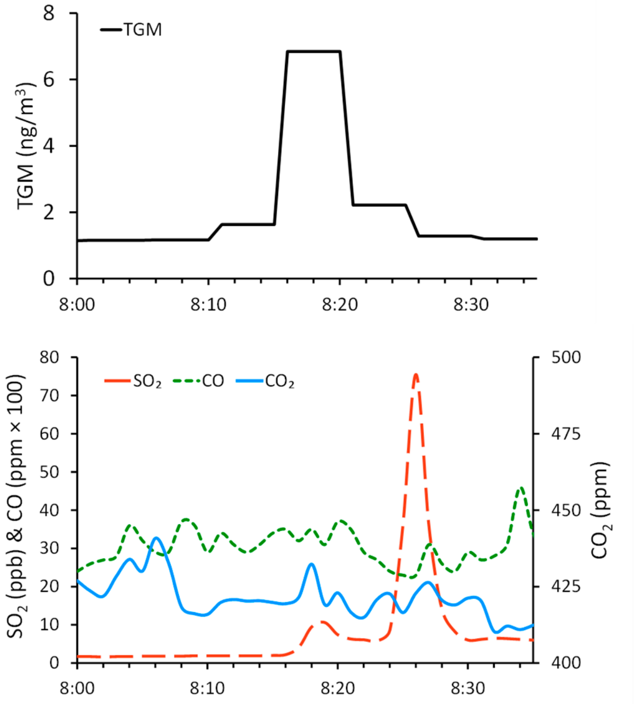

Figure SI-2. 5-min TGM and 1-minute SO_2, CO, and CO_2 measurements on September 5, 2016 from 8:00 MST to 8:35 MST. Note that CO values are shown as ppm×100 to fit the range of values for SO_2 on the primary vertical axis.

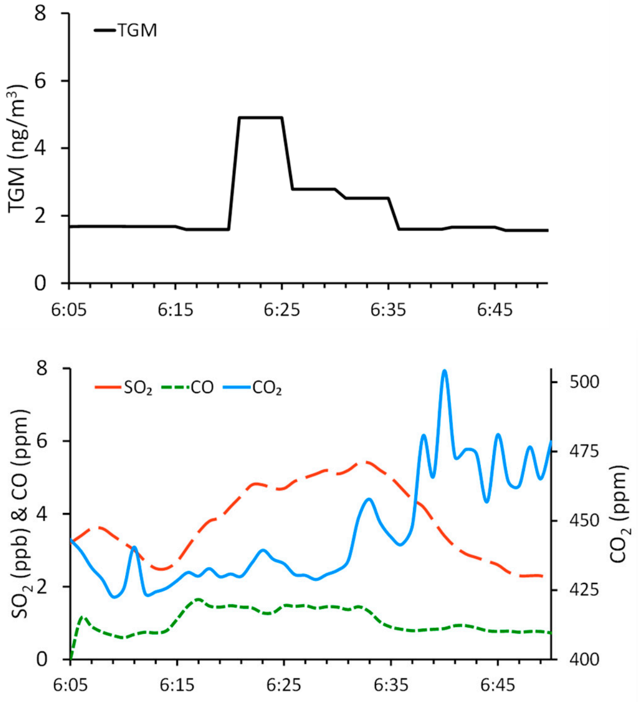

Figure SI-3. 5-min TGM and 1-minute SO_2, CO, and CO_2 measurements on September 27, 2016 from 6:05 MST to 6:50 MST.

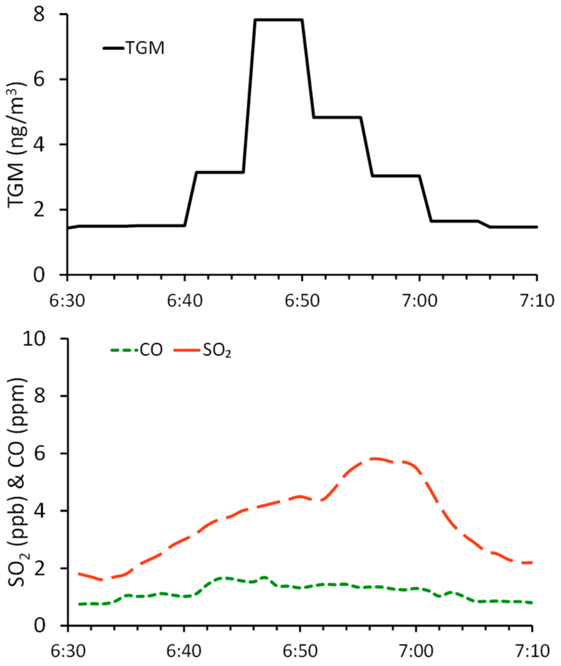

Figure SI-4. 5-min TGM and 1-minute SO_2, CO, and CO_2 measurements on October 10, 2016 from 6:30 MST to 7:10 MST.
